# A shift from Indoor Residual Spraying (IRS) with bendiocarb to Long-Lasting Insecticidal (mosquito) Nets (LLINs) associated with changes in malaria transmission indicators in pyrethroid resistance areas in Benin

**DOI:** 10.1186/1756-3305-6-73

**Published:** 2013-03-16

**Authors:** Razaki A Ossè, Rock Aïkpon, Ghélus Louis Gbédjissi, Virgile Gnanguenon, Michel Sèzonlin, Renaud Govoétchan, Arthur Sovi, Olivier Oussou, Frédéric Oké-Agbo, Martin Akogbéto

**Affiliations:** 1 Centre de Recherche Entomologique de Cotonou (CREC), 06 BP 2604, Cotonou, Benin; 2 Faculté des Sciences et Techniques de l’Université d’Abomey Calavi, Calavi, Benin

**Keywords:** Indoor residual spraying (IRS), Long lasting insecticidal (mosquito) Net (LLIN), *An. gambiae s.l.*, Entomological inoculation rate (EIR), Parity, Blood meal index, Behavior, Benin

## Abstract

**Background:**

Indoor residual spraying (IRS) was implemented in the department of Ouémé-Plateau, southern Benin, in 2008 and withdrawn in 2011, when long lasting insecticidal nets (LLINs) were distributed to the communities that were previously targeted by IRS. Did the LLIN strategy provide a better level of protection against malaria transmission than IRS?

**Methods:**

Entomological surveillance was carried out to assess indicators of transmission risk during the last year of IRS and the first year after the LLIN intervention was put in place (2010–2011). Mosquito biting density was sampled by human landing collection (HLC). Females of *Anopheles gambiae s.l.* were dissected to estimate the parity rates and the blood meal index. A subsample of the *An. gambiae s.l.* collection was tested for presence of *Plasmodium falciparum* sporozoites. In addition, window exit traps and pyrethrum spray catches were performed to assess exophagic behavior of Anopheles vectors.

**Results:**

There were significant increases in all the indicators following withdrawal of IRS. Vector biting density (p<0.001) and longevity (OR=3.81[3.01-4.82] 95% CI; p<0.001) of the *An. gambiae* s.l. increased significantly; so too did the blood meal index (OR=1.48 [1.1-1.99] 95% CI; p<0.001). Entomological inoculation rate, after IRS withdrawal at one surveillance site, Adjohoun, rose two fold (9.0 infected bites/person/9 months (Apr-Dec 2011) versus 3.66 infective bites/person during the 9 months preceding IRS (Apr-Dec 2010). A second site, Missérété, experienced a six-fold increase after IRS cessation (15.1 infective bites/person/9 months versus 2.41 during IRS). Exophily after IRS cessation decreased significantly in all areas (p<0.001) suggesting that mosquitoes were more likely to rest in houses with LLINs, than in houses subjected to IRS.

**Conclusion:**

LLINs did not impact on indicators of transmission to the same levels as did IRS after IRS withdrawal.

## Background

Malaria poses a serious obstacle to development in sub-Saharan Africa [1]. To control transmission, the National Malaria Control Program (NMCP) of Benin relies on an integrated approach to control the intense transmission, characteristic of many programs in this region, through vector-control, early diagnosis and treatment plus prophylaxis during pregnancy.

Malaria vector control is based on two interventions: the long lasting insecticidal (mosquito) net (LLIN) and indoor residual spraying (IRS), both of which have been shown to be effective throughout Africa [2,3]. In Zanzibar, the scale-up of insecticide-treated nets (ITN), indoor-residual spraying (IRS) and Artemisinin combination therapy (ACT) combined reduced malaria-related burden at health facilities by over 75% within 5 years [4]. For example, on Bioko Island, Equatorial Guinea, the simultaneous use of IRS, LLINs and Artemisinin Combination Therapy (ACT) resulted in a 90% drop in the presence of *P. falciparum* circumsporozoite antigen *An. gambiae s.l.* after 4 years. During the same period, malaria parasitaemia in children under 5 years old fell from 42% to 18% and mortality decreased 70% [5]. However, if a country cannot implement both universal IRS and LLINs at the same time, as is the case for most African countries, and makes a decision to withdraw one of the interventions, what would be the result?

The challenge has become sustaining such gains in the face of technical problems, e.g. vector resistance to insecticide, as well as lack of resources for annual renewal of each intervention [6,7]. In recent years, resistance to the pyrethroid insecticides that are used on LLINs, has seriously threatened the efficacy of interventions based on the use of pyrethroid insecticides. N’Guessan *et al*. [8] showed a decrease in the effectiveness of LLINs and IRS, using pyrethroid insecticides, in areas with high levels of vector pyrethroid resistance. Several recent studies conducted in Ouémé Department have showed that *An. gambiae* is highly resistant to pyrethroid and susceptible to bendiocarb [7-9]. The resistance to pyrethroids in our study area is due to the coexistence of metabolic mechanisms and a high frequency of the knockdown resistance mechanism *kdr*[10]. Similar resistance has now been observed across Africa [11-13].

The President’s Malaria Initiative (PMI) of the United States Government, helped the NMCP initiate an IRS program in 2008. The IRS target was in the department of Ouémé-Plateau, where houses in four districts, Adjohoun, Dangbo, Missérété and Sèmè were sprayed with the carbamate insecticide, bendiocarb. In 2008 and 2009, one round of IRS was applied just before the start of the long seasonal rains that usher in the transmission season. In 2010, two IRS rounds were used to extend the IRS protective effect through the second (shorter) rainy season.

IRS was popular and effective. Entomological monitoring, carried out in conjunction with the IRS campaign [14,15], showed a significant drop in the density of female *An. gambiae* s.l. as well as in the entomological inoculation rate (EIR). At the same time, managers of health centers reported a 70% reduction of severe malaria cases.

IRS was withdrawn from Ouémé, and shifted northward to the Department of Atacora in 2011. The technical rationale for the change involved (1) the shorter transmission season in the north, which can be covered by one round of IRS; (2) a high malaria burden and (3) low LLIN coverage when compared with sites in the South [16].

The threat posed by the withdrawal of IRS from Ouémé was addressed by: (1) providing the former IRS target districts with LLINs (universal coverage of 1 net / 2 persons), and (2) continuing the entomological monitoring in the four districts. Monitoring results collected in 2011, following the withdrawal of IRS and distribution of LLINs (LLIN intervention) period, compared with similar results one year earlier, (during IRS) inform this study.

## Methods

### Study area

**Figure 1:**
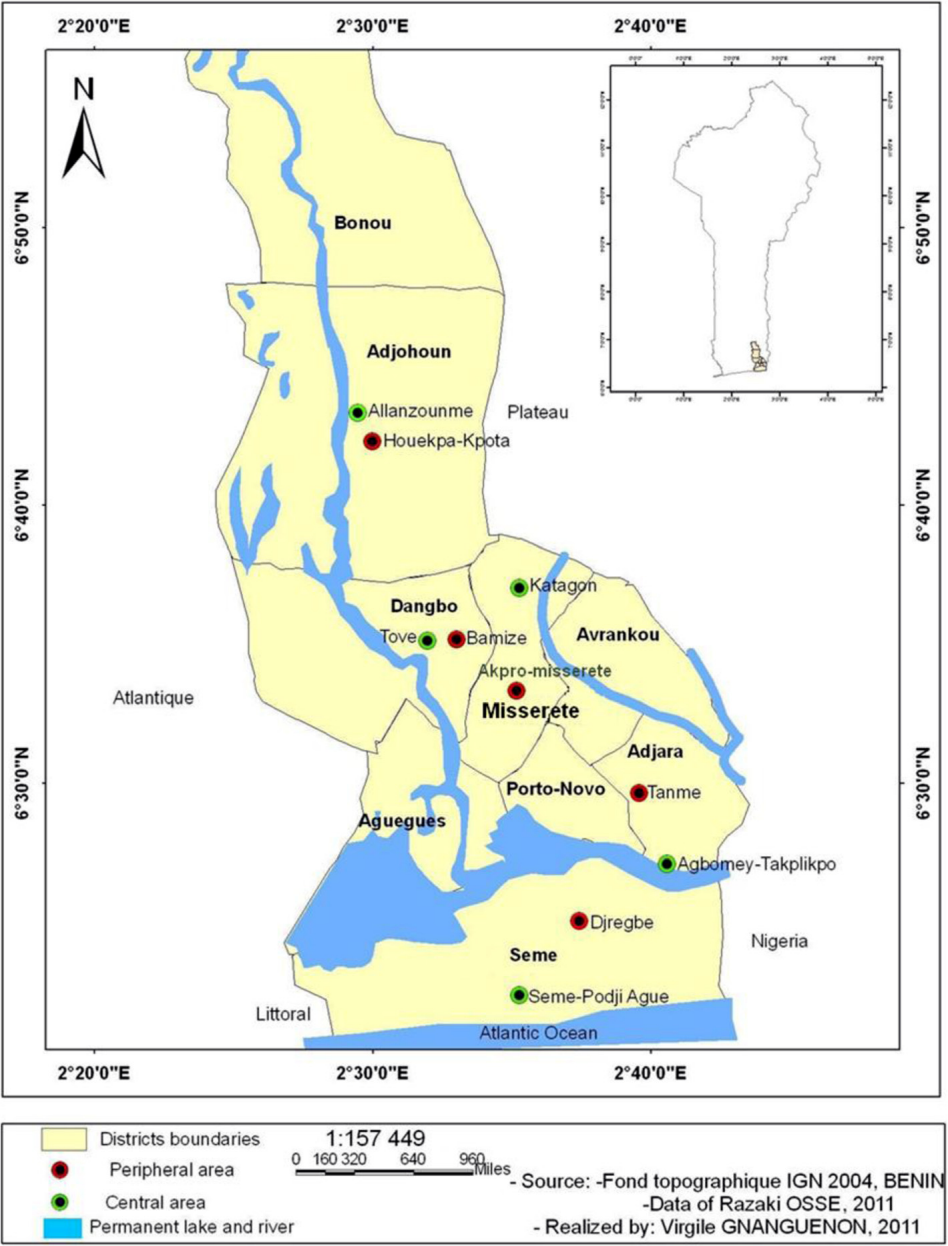
Map of the study area showing localities chosen in central and peripheral areas.

The study area is located in Ouémé Department and includes four districts: Adjohoun, Dangbo, Missérété and Sèmè (Figure 1), an area of approximately 1000 km^2^. The total population is 310,400. There are approximately 65,000 households. In 2010, the incidence of malaria in the Ouémé Department was estimated at 12.6% [17]. Each district includes two different settings: a central area, or ‘plateau’, characterized by the presence of temporary mosquito breeding sites and a peripheral area, or ‘valley’, characterized by the presence of permanent mosquito breeding sites associated with numerous pools and swamps. The distance between the ‘plateau’ and ‘valley’ is higher than two kilometers. The entire region is characterized by a sub-equatorial climate, with two dry seasons (August-September and December-March) and two rainy seasons, April-July (long) and October-November (short). The average annual rainfall is 1500 mm with a relative humidity of 70 ± 5% and an average monthly temperature ranging from 23°C to 32°C.

The data from these four districts previously under IRS intervention were compared with those of a control area (district of Adjara) with the same characteristics but where none of the houses had been sprayed. The 4 districts and the control district are located in the same department, the department of Ouémé. The five areas are populated by the same people, the Ouémènou and Goun, who are mostly traders, artisans and officials.

### IRS Campaigns (2008–2010)

The product chosen by the NMCP to implement IRS in Ouémé was the carbamate class insecticide, bendiocarb. The formulation was 80% WP [18]. The target dosage was 0.4 g a.i./m^2^. In 2008, 2009 and 2010, IRS preceded the long rainy season (March - April). In addition, in 2010, there was a fourth IRS round in August to extend IRS protection throughout the transmission season of the year. The four rounds were performed by volunteers chosen from the local community who were trained by the PMI IRS partner. Each round covered over 90% of the households in the target districts.

### 2011 LLIN distribution

Distribution of LLINs was made by the NMCP in July 2011 and targeted all households in the study area. The coverage of LLINs distributed was 1 LLIN for 1.9 person and proportion of people sleeping under LLIN after the net distribution campaign was 81.76% (Ossè, personal communication). The LLINs, distributed after the withdrawal of IRS, were World Health Organization-approved Olyset® brand nets, rectangular in shape, 150 cm high × 180 cm long × 160 cm wide.

### Entomological monitoring

Monthly mosquito collections occurred at sites located in both the ‘plateau’ and the ‘valley’ ecological zones in all four districts. There were human landing captures (HLCs) on two successive nights from 9:00 p.m to 5:00 a.m, each month, for eighteen (18) months (9 months for each intervention), from April to December 2010 (during the IRS intervention) and from April to December 2011 (after the withdrawal of IRS and the distribution of LLINs). The collection was carried out with an aspirator by adult volunteers who had given their informed consent. In each district, two houses were selected at random for the HLC. In each house, a collector was positioned inside and another outside. Considering the risk of malaria transmission, the collectors were given an antimalarial prophylaxis as a prevention against malaria. During the course of our study all mosquito collectors were monitored for malaria symptoms, which triggered an immediate parasitological test followed by an antimalarial treatment if necessary. Captured female *Anopheles gambiae s.l* were dissected to estimate the percentage of parous mosquitoes. The mosquitoes were then tested to determine the percentage that were positive for sporozoites based on an ELISA test [19].

To determine whether the shift from IRS to LLINs affected vector exophily, exit window traps and morning pyrethrum spray captures (PSCs) were done inside selected houses. Eight exit traps were placed in each district. The exit window traps used were made of a mesh of terylene (synthetic fiber) mounted on a cubical iron frame with an edge measuring 30 cm, with 1 side drawn into a funnel to direct mosquitoes into the trap [20]. The traps were put over the windows of selected houses for two nights per month from 6:00 p.m to 6:00 a.m next day. The houses where the traps were set were selected based on the number of people who slept in them. They were built of mud and wood with a sheet-metal roof and the eve was tight. The area between the upper walls and the roof is closed. Regarding the PSCs, we used the pyrethrum spray Rambo® and white canvas spread on the floor to collect knocked down mosquitoes from 7 a.m. to 9 a.m. The two sampling methods provided independent estimates of the vector density in the houses during IRS use and after its withdrawal as well as the proportion of female mosquitoes exiting from the houses. *Anopheles* mosquitoes collected by these two methods were classified according to the physiological state of their abdomen.

### Processing mosquito collections to estimate biting density, indoor resting density, parity, blood meal index and *P. falciparum* sporozoite rate

Collected mosquitoes were separated into *Culicinae* and *Anophelinae* and then, counted. This was performed using a binocular magnifying glass. The *Anophelinae* were put into cups to be identified to species [21,22]. The ovaries from a certain number of females *An.gambiae s.l.* from human landing catches were dissected to determine parity rates [23]. After this, all *Anopheles gambiae* were stored separately according to the locations they were collected, then labeled and conserved in Eppendorf tubes containing silica gel. The whole ependorf tubes were stored in a freezer at −20°C for future testing by the method of enzyme immunoassay (ELISA CSP) [19].

### Data analysis

Vector density during IRS and after its withdrawal was calculated. Vector biting density, indoor resting density, exophily and the entomological inoculation rates were estimated with confidence intervals at 95%. These rates during the IRS period and after its cessation, the LLIN period, were then compared, using a generalized Poisson model with analysis of deviance of the 2.11.1 version of R software, which takes into account the interdependence of observations in the same area. The proportion of parous females (females that had oviposited at least once) according to the periods was analyzed by logistic regression. The collection of mosquitoes through the windows trap and PSCs was used to estimate the total density of mosquitoes that entered the rooms and the proportion that exited. These data were used to calculate the exophily rate of the mosquitoes (proportion of mosquitoes caught in trap windows by the total number of mosquitoes caught) in each area in 2010 and 2011, as well as their blood feeding rate (number engorged divided by total collected). Significant differences were those with a p-value of <0.05.

### Ethical consideration

Ethical approval for this study was granted by the Ethical Committee of the Ministry of Health in Benin. The mosquito collectors gave prior informed consent and they were vaccinated against yellow fever. They were also subjected to regular medical check-ups with preventive treatments of malaria.

## Results

### Human biting rates (HBR) during the IRS campaign versus HBR following IRS withdrawal and distribution of LLINs

Mosquito biting rates in three of the districts increased after IRS was withdrawn. Also, a significant number of *An. funestus* was collected following the withdrawal of IRS in Missérété ‘valley’ area (14 in 2011 versus 1 in 2010). Table 1 compares *An. gambiae s.l.* biting rates during April - December 2010, during IRS intervention, with similar estimates from April to December 2011, following IRS withdrawal and LLIN installation (Table 1).

**Table 1:** **Human biting rate of *An. gambiae *observed during IRS campaign and following IRS withdrawal**

		**During IRS**				**Following IRS withdrawal**					
		**(Apr.-Dec. 2010)**				**(Apr.-Dec. 2011)**					
		**Total mosquitoes**	**N human catch**	**HBR/ night**	**HBR/ period**	**Total** **mosquitoes**	**N human catch**	**HBR/ night**	**HBR/ period**	**IDR95% IC**	**p-value**
**Adjohoun**											
Central area	Inside	4	28	0.14	38.57	31	34	0.91	246.18	7.75 [6.38-9.42]	<0.001
	Outside	4	28	0.14	38.57	22	34	0.65	174.71	5.50 [4.51-6.71]	<0.001
Peripheral area	Inside	14	28	0.5	135	44	34	1.29	349.41	3.14 [2.81-3.51]	<0.001
	Outside	5	28	0.18	48.21	26	34	0.76	206.47	5.20 [4.35-6.21]	<0.001
**Dangbo**											
Central area	Inside	4	28	0.14	38.57	9	34	0.26	71.47	2.25 [1.81-2.79]	<0.001
	Outside	11	28	0.39	106.07	11	34	0.32	87.35	1 [0.86-1.16]	1
Peripheral area	Inside	70	28	2.5	675	58	34	1.71	460.59	0.83 [0.78-0.88]	<0.001
	Outside	37	28	1.32	356.79	25	34	0.74	198.53	0.68 [0.62-0.74]	<0.001
**Missérété**											
Central area	Inside	3	28	0.11	28.93	14	34	0.41	111.18	4.67 [3.7-5.88]	<0.001
	Outside	14	28	0.5	135	28	34	0.82	222.35	2 [1.78-2.25]	<0.001
Peripheral area	Inside	45	28	1.61	433.93	33	34	0.97	262.06	0.73 [0.68-0.79]	<0.001
	Outside	31	28	1.11	298.93	73	34	2.15	579.71	2.35 [2.18-2.54]	<0.001
**Sèmè**											
Central area	Inside	190	28	6.79	1832.14	51	34	1.5	405	0.27 [0.25-0.28]	<0.001
	Outside	329	28	11.75	3172.5	89	34	2.62	706.76	0.27 [0.26-0.28]	<0.001
Peripheral area	Inside	110	28	3.93	1060.71	58	34	1.71	460.59	0.53 [0.50-0.56]	<0.001
	Outside	217	28	7.75	2092.5	151	34	4.44	1199.12	0.70 [0.67-0.72]	<0.001
**Adjara (Control)**											
Central area	Inside	144	28	5.14	1388.57	6	34	0.18	47.65	0.042 [0.036-0.048]	<0.001
	Outside	137	28	4.89	1321.07	11	34	0.32	87.35	0.08 [0.07-0.09]	<0.001
Peripheral area	Inside	562	28	20.07	5419.29	349	34	10.26	2771.47	0.62 [0.61-0.64]	<0.001
	Outside	521	28	18.61	5023.93	426	34	12.53	3382.94	0.82 [0.8-0.84]	<0.001

*N* number.

*HBR* Human Bite Rate.

*IDR* Incidence Density Ratio.

*IC* Confidence interval.

A significant increase of *An. gambiae* s.l. HBR following the withdrawal of IRS was observed at most sites (p<0.001) (Table 1). In Adjohoun, the average number of indoor bites was 86.78 bites/human/9 months during the IRS campaign against 297.79 after IRS withdrawal (Table 1). In this district, the outdoor aggressive density was significantly higher after IRS withdrawal and LLIN installation (p<0.05) (43.39 bites/human/9 months in April-December 2010 against 190.59 bites/human/9 months after IRS withdrawal). The same observation was made in Missérété and central area of Dangbo (Table 1).

Regarding the overall human biting rate in the districts, in Adjohoun, the *An. gambiae* s.l. biting rate per person increased from approximately 39 bites/person/9 months during the IRS intervention to 210 after IRS withdrawal in the ‘plateau’ area central (p<0.001) and from 92 to 278 bites in the ‘valley’ area. The same kind of increase was observed in Missérété (from 82 to 167 bites/person/9 months after IRS withdrawal in the ‘plateau’ area (p<0.001) and from 366 to 421 bites after IRS withdrawal in the ‘valley’ (p<0.001). On the other hand, while the ‘plateau’ area in Dangbo saw an increase in HBR following withdrawal of IRS (p<0.001) (Table 1).

In the ‘valley’ there was a decrease in HBR following withdrawal. In Sèmè the HBR after IRS withdrawal dropped significantly in both the plateau and valley zones (p<0.001) (Table 1).

### Annual rainfall confounds interpretation of HBR data

**Figure 2:**
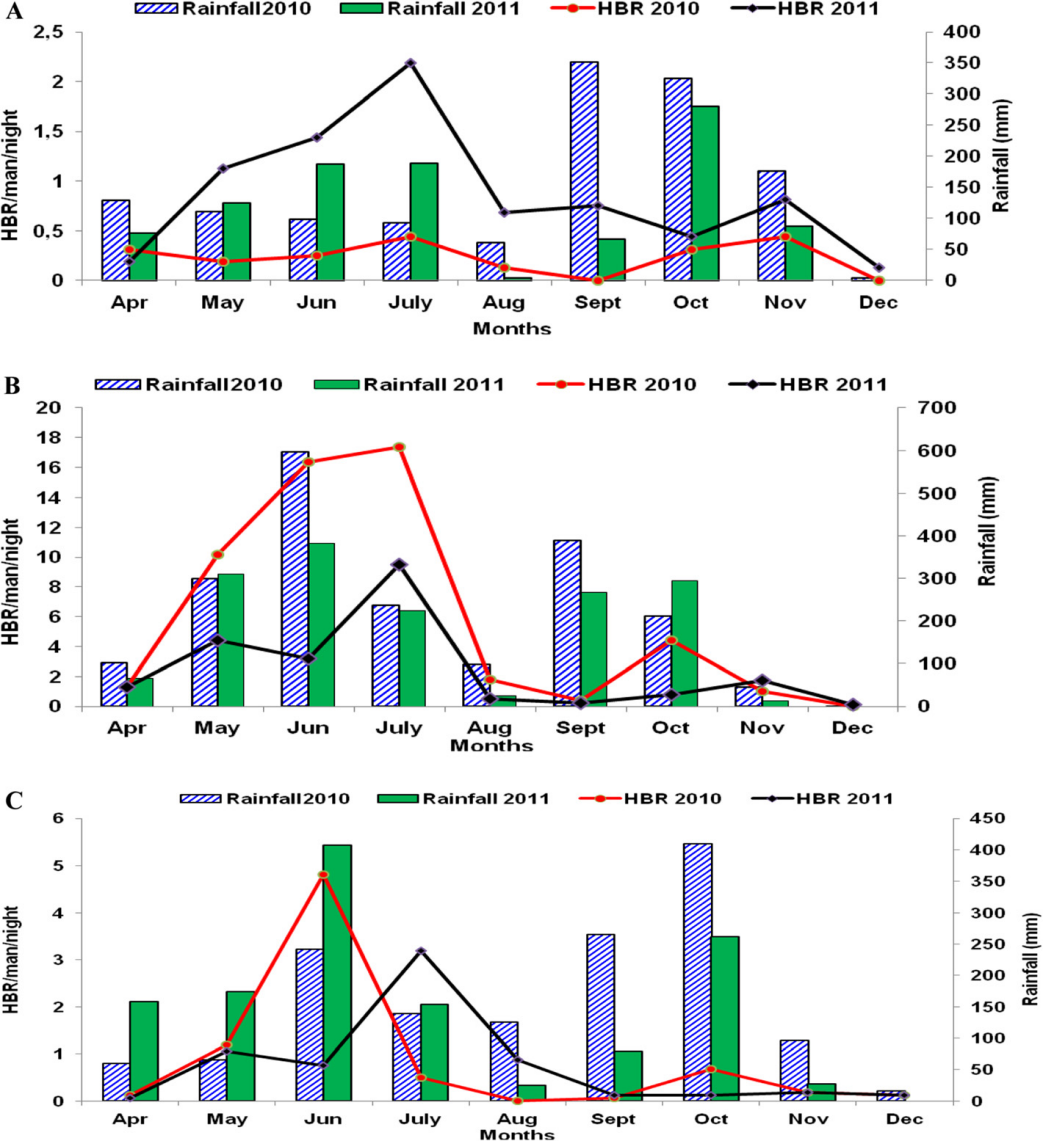
**Aggressive variation of *An. gambiae *s.l density depending on rainfall in Adjohoun (A), Sèmè (B) and Dangbo (C).**

The highest anopheline densities were observed during the long (Apr - Jul) and the short (Oct – Nov) rainy seasons. However, annual differences in rainfall also affected HBR estimates. The high rainfall in Sèmè in 2010 justified the decrease of *An. gambiae* HBR from 1.31 bites/person to 17.38 in July 2010 against 1.31 to 9.5 in July 2011 (after IRS cessation) (Figure 2B). The high rainfalls in 2010 washed away some breeding sites of *An. gambiae* larvae. Moreover, the rainfalls recorded by ASECNA (Agency for Aerial Navigation Safety in Africa and Madagascar) in 2011 in Adjohoun during the same period were higher than in 2010 (Figure 2A). In return, the HBR obtained in 2011 (after IRS cessation) was higher than that recorded in 2010 (during IRS intervention) (0.19 to 2.18 in July 2011 against 0.31 to 0.44 in July 2010).

### *An. gambiae s.l.* exophily reduced after IRS withdrawal

**Figure 3:**
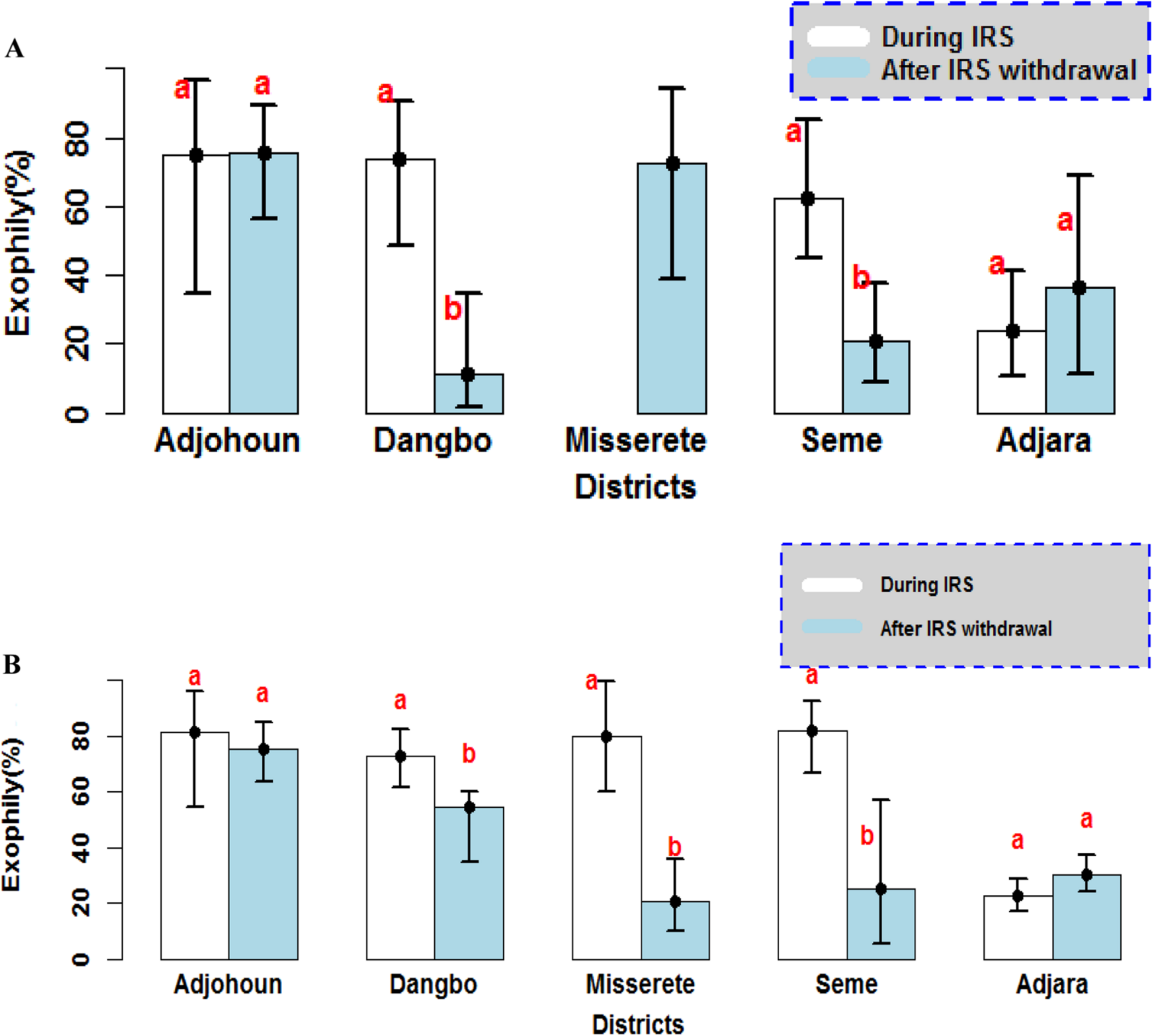
**Exophily rate of *An. gambiae *observed during IRS campaign and after IRS withdrawal in central area (A) and peripheral area (B) in the various districts.** Exophily rate carrying the same letter per district was not significantly different (p> 0.05).

Figure 3 shows *An. gambiae* exophily during the IRS intervention period and after its withdrawal. While exophily did not show a significant change after IRS withdrawal in Adjohoun, in the other three districts, a significant decrease was observed (p<0.001) (Figure 3A and B). In Dangbo, the exophily rate decreased from 73% during IRS intervention to 44% after its withdrawal. The exophily rate in Missérété was 80% during IRS and 31% after withdrawal. Similarly, the exophily rate in Sèmè fell from 79% during IRS to 22% after withdrawal.

### *Anopheles gambiae* s.l longevity increases after IRS withdrawal

**Figure 4:**
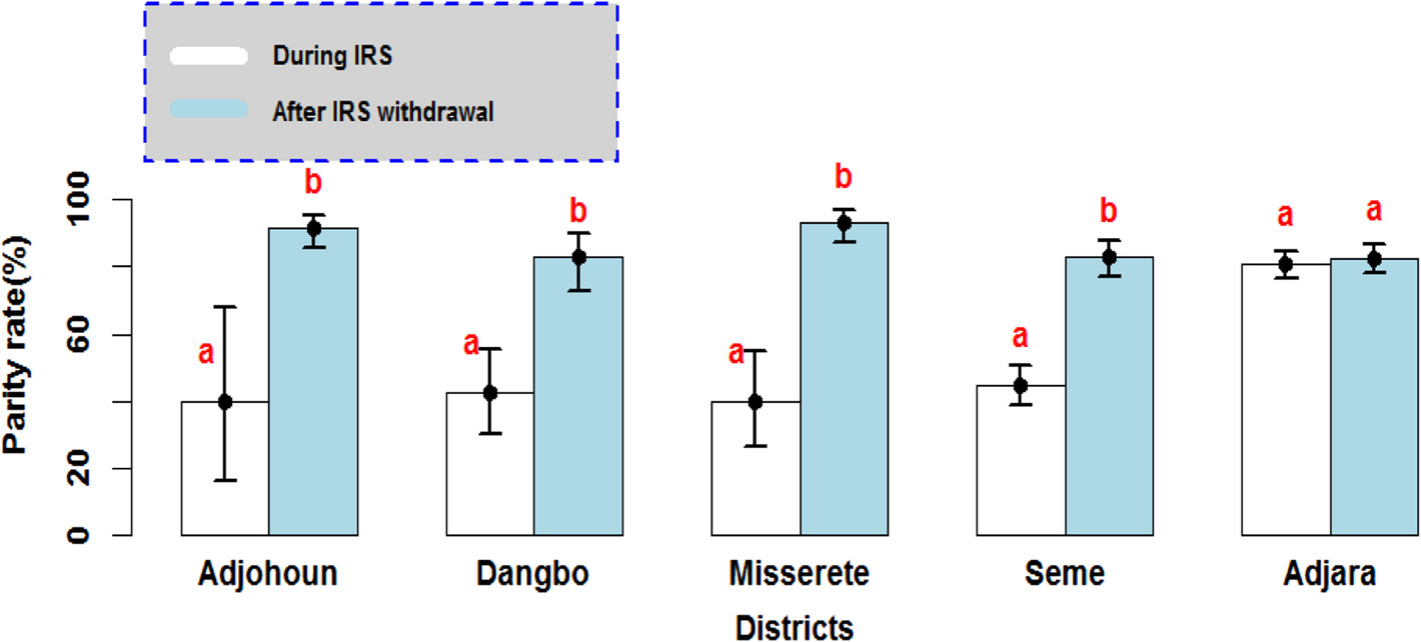
**Parity rate of *Anopheles gambiae *observed during the implementation of IRS and after IRS withdrawal.** The rate carrying the same letter per district was not significantly different (p> 0.05).

A total of 1681 *Anopheles* were dissected to estimate physiological age. The proportion of older female mosquitoes, e.g. those that had oviposited at least once, was significantly higher after IRS withdrawal in all four districts (OR = 3.81[3.01-4.82] 95% CI; p<0.001). During IRS, the parity rate of *An. gambiae* s.l. observed in Adjohoun, Dangbo, Missérété and Sèmè was respectively 40%, 42%, 45% and 40%. However, after IRS withdrawal, parity rates increased: 92%, 88%, 93% and 83% respectively (Figure 4).

In the control area, on the other hand, the parity rate was the same in both periods (OR = 1.13 [0.77-1.65] 95% CI; p = 0.54).

### Vector blood meal index (BMI) inside the houses increases after IRS withdrawal

**Figure 5:**
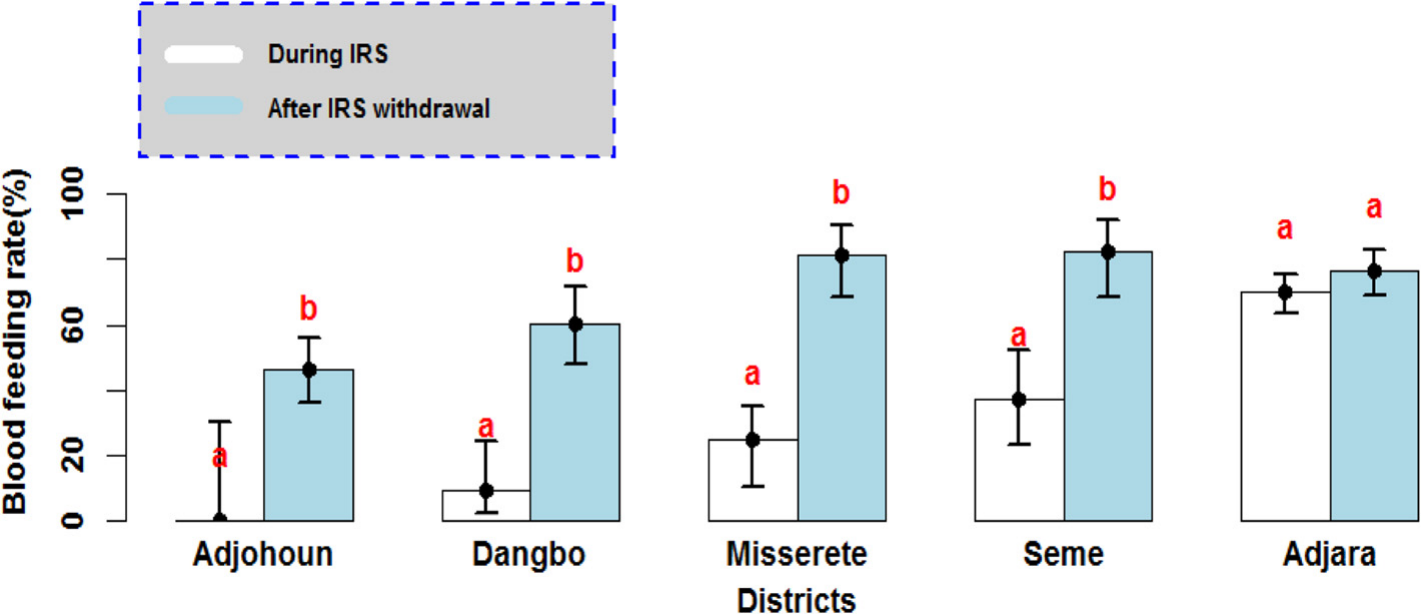
**Blood feeding rate of *Anopheles gambiae *observed during IRS implementation and after IRS withdrawal.** The rate carrying the same letter per district was not significantly different (p> 0.05).

The blood feeding rate of *An. gambiae* was respectively 0%, 9%, 25% and 37% in Adjohoun, Dangbo, Missérété and Sèmè during IRS (Figure 5). BMI increase after IRS withdrawal in all districts (OR = 1.48 [1.1-1.99] 95% CI; p <0.001) (46% in Adjohoun, 60% in Dangbo, 81% in Missérété and 83 in Sèmè).

**Figure 6:**
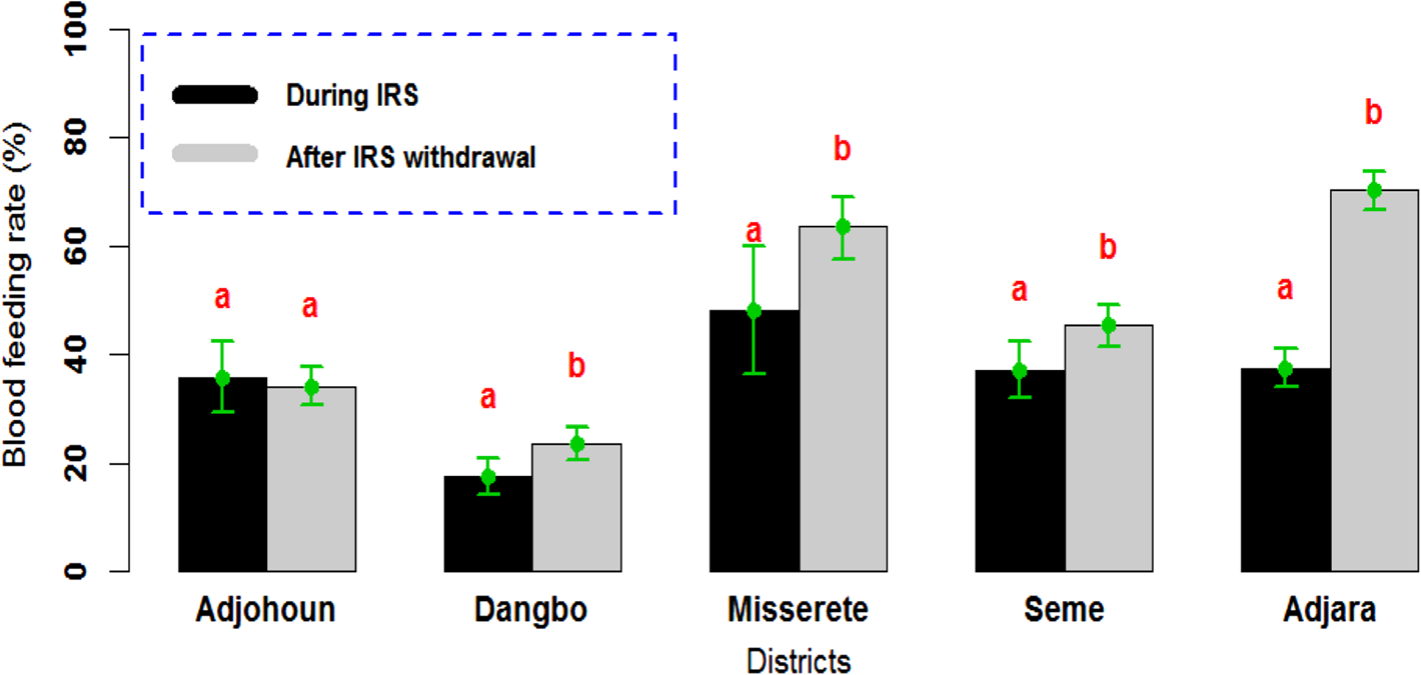
**Blood feeding rate of Culicidae observed during IRS implementation and after IRS withdrawal.** The rate carrying the same letter per district was not significantly different (p> 0.05).

All mosquitoes showed the same phenomenon: the blood feeding rate obtained after IRS cessation was significantly higher than that recorded during IRS intervention in Dangbo, Missérété and Sèmè districts (OR = 1.65 [1.46-1.86] 95% CI; p <0.001). No significant difference was observed in Adjohoun between blood feeding rates in both periods (OR = 0.93 [0.67-1.27] 95% CI; p = 0.64) (Figure 6).

### Change in EIRs after IRS withdrawal

During the IRS intervention period, 493 heads and thoraxes from a random selection of females *An. gambiae s.l*. were tested using ELISA CSP to identify the presence of sporozoites. Ten (2.0%) tested positive for *P. falciparum* sporozoite antigen. Nine out of the ten that tested positive were collected in April and May during the third IRS campaign. But just a single infected mosquito was caught during the fourth round of IRS (three months later - Aug) (Table 2).

**Table 2:** **Entomological inoculation rate (EIR) of *An. gambiae *observed during IRS campaign and following IRS withdrawal**

		**During IRS**				**Following IRS withdrawal**					
		**(Apr.-Dec. 2010)**				**(Apr.-Dec. 2011)**					
**Localities**	**Variables**	**3**^**rd **^**round of IRS**	**4**^**th **^**round of IRS**	**Total**	**EIR/period**	**April-July**	**Aug.-Dec.**	**Total**	**EIR/period**	**IDR**	**Comparison EIR/period**
		**(Apr.-Jul.)**	**(Aug.-Dec.)**	**(Apr.-Dec.)**				**(Apr.-Dec.)**		**95% IC**	
Adjohoun central area	Thorax	4	4	8	0	25	16	41	5.2	∞	p<0.001
	Thorax+	0	0	0		1	0	1			
	Is	0	0	0		0.04	0	0.02			
	EIR	0	0	0		0.04	0	0.02			
Adjohoun peripheral area	Thorax	15	10	25	7.33	39	27	66	12.83	1.75 [1.72-1.78]	p<0.001
	Thorax+	2	0	2		2	1	3			
	Is	0.13	0	0.08		0.05	0.04	0.05			
	EIR	0.07	0	0.03		0.07	0.03	0.05			
Dangbo central area	Thorax	13	2	15	0	11	9	20	0	1	p=1
	Thorax+	0	0	0		0	0	0			
	Is	0	0	0		0	0	0			
	EIR	0	0	0		0	0	0			
Dangbo peripheral area	Thorax	93	14	107	19.29	48	17	65	5.23	0.27 [0.27-0.28]	p<0.001
	Thorax+	3	1	4		0	1	1			
	Is	0.03	0.07	0.04		0	0.06	0.02			
	EIR	0.11	0.04	0.07		0	0.02	0.02			
Missérété central area	Thorax	12	5	17	0	38	16	54	3.09	∞	p<0.001
	Thorax+	0	0	0		1	0	1			
	Is	0	0	0		0.03	0	0.02			
	EIR	0	0	0		0.02	0	0.01			
Missérété peripheral area	Thorax	44	32	76	4.82	45	58	103	27.07	5.61 [5.53-5.69]	p<0.001
	Thorax+	1	0	1		4	2	6			
	Is	0.02	0	0.01		0.09	0.03	0.06			
	EIR	0.04	0	0.02		0.15	0.04	0.1			
Sèmè central area	Thorax	80	58	138	36.27	121	9	130	25.85	0.71 [0.71-0.72]	p<0.001
	Thorax+	2	0	2		6	0	6			
	Is	0.03	0	0.01		0.05	0	0.05			
	EIR	0,41	0	0.13		0.2	0	0.1			
Sèmè peripheral area	Thorax	73	34	107	14.73	166	43	209	7.44	0.5 [0.5-0.51]	p<0.001
	Thorax+	1	0	1		2	0	2			
	Is	0.01	0	0.01		0.01	0	0.01			
	EIR	0.13	0	0.05		0.06	0	0.03			
Adjara central area	Thorax	44	83	127	96.01	15	2	17	8.1	0.08 [0.04-0.17]	p<0.001
	Thorax+	3	6	9		2	0	2			
	Is	0.07	0.07	0.07		0.13	0	0.12			
	EIR	0.45	0.25	0.36		0.06	0	0.03			
Adjara peripheral area	Thorax	120	104	224	536.15	516	257	773	124.2	0.23 [0.19-0.28]	p<0.001
	Thorax+	14	9	23		23	6	29			
	Is	0.12	0.09	0.1		0.04	0.02	0.04			
	EIR	3.83	0.5	1.99		0.61	0.17	0.46			

Thorax +: positive thorax.

*Is* sporozoïtique.

*EIR* Entomological Inoculation Rate (infected bite/man/night).

*IDR* Incidence Density Ratio.

*IC* Confidence interval.

On the other hand, 20 heads and thoraxes out of 688 (2.9%) tested positive after IRS withdrawal (p = 0.330).

With the infected mosquitoes collected in April 2010, at the beginning of the third round of IRS, the results show that only one mosquito out of 159 was infected during IRS intervention (Is=0.0063) and four out of 195 after IRS withdrawal (p= 0.233).

In the control area (Adjara), the sporozoite index was high during both periods: 0.091 (32/351) in 2010 and 0.039 (31/790) in 2011.

No difference was observed in sporozoite rates before versus after IRS (data from all sites combined). It is estimated that during the IRS intervention period, each inhabitant of the four districts would receive an average of 13 infected bites over a period of nine months (April –December 2010). A similar rate was observed after IRS cessation with 10.40 infected bites during the same period (Table 2). When these data are examined by site, the EIR in Adjohoun and Missérété after IRS withdrawal was higher than that observed during the IRS intervention period (9 infected bites/person in Adjohoun after IRS withdrawal against 4 during IRS intervention period; in Missérété, 15 infected bites/person after IRS withdrawal against 3 during the intervention). But it was rather the contrary that was observed in Sèmè and Dangbo after IRS cessation (Table 2).

## Discussion

Difficult decisions, such as the one to withdrawal IRS from an area, must be taken by National Malaria Control Programs when faced with demands that exceeds the availability of resources for control. Providing an alternative, proven and effective vector control intervention, such as LLINs, to replace IRS can improve, albeit not completely, the negative consequences of IRS withdrawal. The challenge is especially great in situations like Ouémé Plateau District, where the transmission season is long and the entomological inoculation rate is high [24]. Evaluating the impact of IRS withdrawal and replacement with high coverage LLINs to inform this issue is an important objective of entomology monitoring and evaluation. The example of IRS withdrawal in Southern Benin provides a good case in point. It is costly, and difficult to implement IRS without assistance from external partners. Nonetheless, this intervention is highly effective and requires little in terms of compliance from the community. The problem with IRS is; can it be sustained independently? In the case of Ouémé there was a concerted effort to document the impact of LLINs and to reissue the population of Ouémé department, as well as the NMCP that the alternative, universal coverage with LLINs following withdrawal of IRS, would provide a similar level of protection from malaria.

However, in this case, entomological indicators of malaria transmission increased significantly after IRS withdrawal /LLIN distribution. LLINs are proven and effective, however impact also depends on the existence of a strong ‘net culture’ in the community. For example, proper use (compliance with nightly use of sleeping under nets) and care of nets is a key behavior change that must take place if LLIN interventions are to be as effective as IRS. High coverage with LLINs is also necessary. Unfortunately, in this study there was no assessment of LLIN use at the community level to evaluate how well communities adapted to the unique aspects of the LLIN.

This study suggests that in Ouémé department multiple entomologic measures of malaria transmission increased, raising the risk of transmission, at a time when a new intervention, universal coverage with LLIN, was introduced, after IRS withdrawal. The HBR increased following withdrawal of IRS and hanging of LLINs could not initially be kept during the IRS campaign despite the distribution of treated nets to every household. Vector biting density increased in three of the assessment districts after withdrawal of IRS (Adjohoun, Dangbo and Missérété). The lower HBR observed in Sèmè after withdrawal, an exception to the pattern, could be explained by the fact that the breeding sites and the *An. gambiae* larvae had been washed away by waters from the heavy rains in that district in 2011. Such a high rainfall was not observed in the other districts. The HBR drop also observed after IRS withdrawal in the control area, was probably due to a higher rainfall in 2010 than in 2011. This reduction of HBR obtained in the control area may be due not only to changes in rainfall but also the increase of LLIN coverage, which could contribute to the reduction of HBR and the sporozoite rate during the two periods. It can be concluded that the withdrawal of IRS and distribution of LLINs is associated with an increase in HBR.

HBR in Sèmè decreased after withdrawal, which contradicts the pattern in the other sites. However, rainfall in this district was lower after withdrawal than before. We speculate that this shift in rainfall, not seen at the other sites, may be responsible for the HBR decrease following withdrawal.

An increase in malaria transmission was observed in the districts after IRS withdrawal. In Adjohoun and Missérété, the entomological inoculation rate obtained after IRS withdrawal (9 months) was significantly higher (p<0.001) than that obtained during IRS. Such a variation in the transmission level is probably due to the high *An. gambiae* HBR and the large CSP index recorded after IRS withdrawal.

Compared to Adjohoun, Dangbo and Missérété, malaria transmission remained high in Sèmè during both periods. But the EIR in this area after IRS cessation was less than that recorded before withdrawal of IRS. This difference may reflect the high Culicidae nuisance problem in this area. If nuisance biting was exceptionally high, compliance with nightly use of nets may have been better that in other sites. Indeed, after IRS cessation, the use of LLINs proved successful in reducing malaria transmission in this area of high anopheline aggressiveness. This confirms the work of Kelly-Hope *et al*. [2] and Akogbéto *et al.*[14], that nuisance biting improves compliance with LLIN use. Moreover, the results of research in Africa showed that vector control by widespread use of LLINs is a strategy that can reduce malaria morbidity by 50 to 60% and overall mortality by 20% [4,25,26].

It is also important to mention some of the limits of this study namely, the difficulty of comparing longitudinal data without a proper control area where IRS would have been maintained to control for any confounding factors such as climatic conditions or the use of mosquito nets.

The presence of Olyset nets in houses after IRS cessation did not increase the exit rate of *An. gambiae* and *Culex spp* at all. These results show that the excito-repellent effect of permethrin on *An. gambiae* was not remarkable, probably because of the resistance of this vector to pyrethroids. These results are reminiscent of those from N’Guessan *et al*. [8], which showed a decrease in the effectiveness of pyrethroid-treated nets, especially in Benin.

## Conclusion

After IRS withdrawal in Ouémé, an alternative vector control intervention, LLINs, was provided to all households in the former IRS target area. However, entomological monitoring and evaluation results for the year prior to withdrawal and the year afterwards, indicate that LLINs did not impact indicators of transmission to the same level as did IRS. Instead a significant increase in several measures of transmission was observed following IRS withdrawal in most of the sites. The human biting rate increased in Adjohoun, Dangbo and Missérété but decreased in Sèmè. Also, the decrease in transmission, observed in the control area (Adjara), reflected a rainfall pattern than was not characteristic of the other sites. In summary, IRS cessation resulted in EIR increase in three of the districts, a decrease in *An. gambiae* exophily, and increased vector longevity (parity). The overall increase in measures of transmission observed following withdrawal of IRS and introduction of LLINs points to a need for information, education and communication activities at the community level in order to reduce the risk of increasing transmission that may accompany similar changes in vector control strategy. Furthermore there is a need for ongoing monitoring of transmission, using entomological techniques, following withdrawal to IRS in order verify impact and avoid resurgence of malaria due to differences in the impact of LLINs on transmission.

## Competing interests

The authors declare that they have no competing interests.

## Authors’ contributions

RO, RA, GG and MA designed the study. RO, VG, RG, AS and OO carried out the field activities. RO and FO analyzed the data. RO drafted the manuscript. MA, MS, RO and RA critically revised the manuscript for intellectual content. All authors read and approved the final manuscript.
